# Prolonged grief, depression, and posttraumatic stress in disaster-bereaved individuals: latent class analysis

**DOI:** 10.1080/20008198.2017.1298311

**Published:** 2017-03-10

**Authors:** Lonneke I. M. Lenferink, Jos de Keijser, Geert E. Smid, A. A. A. Manik J. Djelantik, Paul A. Boelen

**Affiliations:** ^a^ Department of Clinical Psychology and Experimental Psychopathology, Faculty of Behavioural and Social Sciences, University of Groningen, Groningen, the Netherlands; ^b^ Department of Clinical Psychology, Faculty of Social Sciences, Utrecht University, Utrecht, the Netherlands; ^c^ Foundation Centrum ’45, Diemen, the Netherlands; ^d^ Arq Psychotrauma Expert Group, Diemen, the Netherlands

**Keywords:** Bereavement, loss, disaster, trauma, stress, resilience, unrealness

## Abstract

**Background**: Hundreds of individuals lost one or more significant others in the MH17 plane crash in 2014 in Ukraine. The current study is the first to explore subgroups of disaster-bereaved individuals based on presence of psychopathology clusters. This may inform the development of diagnostic instruments and tailored interventions.

**Objective**: Aims of the current study were to examine (1) subgroups based on presence of prolonged grief disorder (PGD), major depressive disorder (MDD), and posttraumatic stress disorder (PTSD) symptom clusters and (2) associations between class membership, disaster-related variables (i.e. experiencing multiple losses, conducting multiple burials for the same deceased, and time to confirmation of death), and a sense of unrealness.

**Method**: Self-rated PGD (10 items of the Traumatic Grief Inventory represented in two symptom clusters), MDD (16-item Quick Inventory Of Depressive Symptomatology represented in one symptom cluster), and PTSD (20-item PTSD Checklist for DSM-5 represented in four symptom clusters) from 167 participants were subjected to latent class analysis to identify subgroups (i.e. classes). Correlates of class membership were assessed using the three-step approach.

**Results**: A three-class solution yielded the best model fit. Class 1 (Resilient class; 20.0%) was predominantly characterized by low probability of PGD, MDD, and PTSD symptom clusters, class 2 (PGD class; 41.8%) by moderate to high probability of presence of PGD, and class 3 (Combined class; 38.2%) by moderate to high probability of presence of PGD, MDD, and PTSD symptom clusters. Compared with the Resilient class, a sense of unrealness was more likely to be experienced by individuals in the PGD class and the Combined class.

**Conclusions**: Our results indicate that subgroups of disaster-bereaved individuals can be distinguished based on the presence of PGD, MDD, and PTSD symptom clusters. A sense of unrealness was the strongest distinguishing feature of the subgroups.

## 1. Background

On 17 July 2014 flight MH17 from Amsterdam to Kuala Lumpur crashed in Ukraine due to a missile impact. All 298 passengers, including 193 Dutch citizens, died (Dutch Safety Board, 2015). Worldwide, many individuals have lost their lives in disasters. For example, more than 227,000 people died due to the Indian Ocean tsunami in 2004 (Johannesson, Lundin, Hultman, Fröjd, Michel, 2011) and about 2800 people died in the 9/11 attacks in 2001 (Perlman et al., 2011). Explorations of psychopathology post-disasters have primarily focused on posttraumatic stress disorder (PTSD) in adults directly exposed to the disaster (e.g. as a survivor) and to a lesser extent on individuals who lost a significant other due to a disaster (Galea, Nandi, & Vlahov, 2005; Perlman et al., 2011).

The current study focuses on patterns and correlates of psychological symptoms in individuals who lost one or more significant others in the Ukrainian plane crash. This is important for a number of reasons. Firstly, the likelihood of developing psychopathology may be higher for manmade disaster-bereaved individuals compared with individuals confronted with non-violent loss (e.g. due to illness). Specifically, the violent nature of the loss in the context of manmade disaster (Galea et al., 2005) and suffering multiple simultaneous losses seems to be linked to increased psychopathology levels (Kristensen, Weisaeth, & Heir, 2010). Gaining more knowledge about disaster-related risk factors may help to identify individuals at risk for development of psychopathology.

Secondly, different studies have explored the prevalence and correlates of psychopathology following disaster-related loss, including the 9/11 attacks (Bonanno, Galea, Bucciarelli, & Vlahov, 2006) and the Indian Ocean tsunami (Kristensen et al., 2010). However, to our knowledge, no studies have yet examined whether subgroups can be distinguished among disaster-bereaved individuals in terms of different forms of psychopathology, including prolonged grief disorder (PGD), major depressive disorder (MDD), and PTSD. PGD shows overlap with, yet is distinguishable from, MDD and PTSD (Boelen, van de Schoot, van den Hout, de Keijser, & van den Bout, 2010). The most prominent difference between these three syndromes is that PGD is dominated by yearning for the deceased, while MDD is characterized by anhedonia and dysphoria, and PTSD is dominated by intrusion and hyperarousal symptoms (Maercker & Znoj, 2010; Prigerson et al., 2009). Treatment effects may differ between subgroups of bereaved individuals that are characterized by different symptom-profiles (Smid et al., 2015). Identifying subgroups may provide valuable information for the development of diagnostic instruments and tailored interventions (Rosner, 2015).

The first aim of this study was to explore subgroups (i.e. latent classes) based on endorsement of PGD, MDD, and PTSD symptoms in manmade disaster-bereaved individuals, using latent class analysis (LCA). In recent years, there is growing interest in these person-centred analyses of responses to adverse life events (Armour et al., 2015; Cloitre, Garvert, Weiss, Carlson, & Bryant, 2014; Contractor et al., 2015). LCA identifies unobserved subgroups of individuals based on predefined indicators (in the current study presence of PGD, MDD, and PTSD symptom clusters). Previous LCA studies in bereaved samples were either focused on PGD and PTSD symptoms (Nickerson et al., 2014) or PGD and MDD symptoms (Boelen, Reijntjes, Djelantik, & Smid, 2016). These studies indicated that three to four classes can be distinguished: (1) a Resilient class, (2) a PGD class, (3) a PGD combined with MDD or PTSD class, and (4) in the study of Nickerson et al. (2014) also a distinct PTSD class. To the best of our knowledge, the current study is the first to explore classes based on PGD, MDD, and PTSD assessed in a single study. Based on previous findings (Boelen et al., 2016; Nickerson et al., 2014) we expected to identify three classes: a Resilient class, PGD class, and a Combined class of individuals who experience comorbid symptoms. We did not expect to identify a distinct PTSD class because, unlike our sample, Nickerson et al.’s (2014) sample consisted of bereaved refugees who were also exposed to other traumatic events.

The second aim of the current study was to explore associations between class membership on the one hand and sociodemographic variables and disaster-related variables on the other hand. Previous research among disaster-bereaved individuals has shown that psychopathology levels were higher among women, more recently (compared with remotely) bereaved individuals, and those with closer kinship to the deceased (Kristensen, 2010; Li, Chow, Shi, & Chan, 2015). We therefore expected that these variables would distinguish the Resilient class from the other classes. Disaster-related variables included: experiencing multiple losses, conducting multiple burials for remains of the same deceased, and time to confirmation of death. Kristensen et al.’s (2010) study showed that suffering multiple disaster-related losses concurrently seems to be associated with elevated psychopathology levels. In contrast, other studies showed that the effect of multiple losses disappeared when the nature of the relationship to the deceased was taken into account (Li et al., 2015; Stammel et al., 2013). Kristensen et al. (2010) also found longer time to confirmation of death to be associated with elevated psychopathology levels. Once confirmation of death has been received, grief rituals that may facilitate adjustment to the loss, such as a funeral (Castle & Phillips, 2003), can be conducted. However, many bereaved individuals losing loved ones in the Ukrainian plane crash received remains of their deceased loved one at different time points. As a result, they were brought into the position where they could bury remains of their loved ones at more than one occasion. These disaster-related variables may fuel a subjective sense that the loss ‘feels’ surreal, as if it did not happen, despite knowing that it did – a phenomenon that has been referred to as ‘a sense of unrealness’ before (Boelen, 2010). Boelen (2010) proposed that a sense of unrealness is the explicit equivalent of the implicit process that is defined as “poor integration of the loss into autobiographical knowledge” (pp. 239). Adaptation to loss goes hand in hand with integration of the irreversibility of the loss into the autobiographical knowledge base. Over time then, confrontation with reminders of the loss becomes less disturbing. In contrast, poor integration of the loss may lower the threshold of feeling shocked about the loss, once the bereaved is confronted with loss-related stimuli (Boelen, 2010). This sense of unrealness is proposed to be one of the key processes that exacerbate or maintain PGD symptoms (Boelen, 2010; Boelen, van den Hout, & van den Bout, 2006). Although a sense of unrealness shows some overlap with PGD symptoms (especially the symptom “difficulty accepting the loss”), confirmatory factor analysis has shown that unrealness and PGD are distinct phenomena (Boelen, 2010). We hypothesized that a sense of unrealness would be positively associated with membership of classes displaying more pervasive psychopathology compared with individuals in the Resilient class.

Finally, we examined the association between functional impairment and class membership. Based on Kristensen, Weisaeth, Hussain, & Heir (2015) we expected that functional impairment levels would be higher in the psychopathology classes compared with the Resilient class.

## 2. Methods

### 2.1. Participants

Data were used from 167 individuals taking part in the first assessment of an ongoing longitudinal study among people who lost loved ones in the Ukrainian plane crash. Data collection took place between May 2015 and January 2016. In total 193 individuals started the survey, but 26 participants did not complete the survey and, as a result, did not complete the PGD, MDD, and PTSD measures and were therefore excluded from the analyses. Participants who did not complete the survey were asked by telephone or e-mail why they stopped. The major reason was that they did not know the date of birth or identification of their lost loved one(s) and they were therefore not able to continue the online survey due to the forced response format. Completers and non-completers did not significantly differ with respect to gender, age, educational level, time since loss, number of losses, and relationship to the deceased.

### 2.2. Procedures

Potential participants were invited to take part in the online survey study. In case a participant preferred a paper-and-pencil survey, this was sent by regular mail together with a stamped return envelope (*n* = 26). Participants were recruited along different pathways. Invitation letters or emails were sent to 149 members of the MH17 Disaster Foundation (a Dutch support organisation for bereaved of the Ukrainian plane crash). An announcement was placed on a Dutch webpage with information about the disaster, accessible for approximately 450 bereaved individuals. Victim Support the Netherlands (a governmental organisation offering practical and legal support to victims of loss and trauma) contacted 166 spokespersons of families by letter or telephone to invite them to participate. Potential participants were also recruited via presentations at support organizations and through media attention. Lastly, individuals who signed up for the study were asked to invite others.

In total, 69 participants (41.3%) were recruited via Victim Support, 46 (27.5%) via the MH17 Disaster Foundation, 42 (25.1%) via referral by an acquaintance, and 10 (6.0%) otherwise. An approximate indication of the response-rate is 36.3% (41.6% for Victim Support and 30.9% for the MH17 Disaster Foundation; the response-rate for the other sources is unknown). Ethical approval for this study was obtained from a local ethical board. Informed consent was obtained from all participants.

### 2.3. Measures

#### 2.3.1. Indicators

PGD symptoms were assessed with the 18-item Traumatic Grief Inventory (Boelen & Smid, in press). In the current study, 10 items of this measure were used, resembling proposed criteria for PGD (Prigerson et al., 2009) that will likely be included in the forthcoming edition of the International Classification of Diseases (ICD). If the participant had experienced multiple losses, he/she was instructed to fill in the PGD measure while keeping in mind the loss that was most often on his/her mind and/or was experienced as most stressful. If participants felt unable to choose, they could fill in the measure multiple times. The PGD measure with the highest sum score was used in the analyses. Following previous LCA-studies (Boelen et al., 2016; Nickerson et al., 2014), each item (range 1–5) rated as 3 (‘sometimes’), 4 (‘frequently’), or 5 (‘always’) was considered as a symptom endorsed. Then, following the diagnostic scoring rule of PGD (Prigerson et al., 2009), the 10 items were divided over two indicators in the LCA as follows: endorsement of the ‘yearning’ item was used as indicator of the presence of ‘Separation distress’ and endorsement of at least five of nine other PGD symptoms (i.e. ‘Confusing about one’s role in life’, ‘Difficulty accepting death’, ‘Avoidance of reminders of the loss’, ‘Difficulty trusting others’, ‘Bitterness or anger’, ‘Difficulty moving on’, ‘Numbness’, ‘Feeling life is meaningless’, and ‘Feeling stunned’) was used as indicator of the presence of the symptom cluster ‘Cognitive, emotional, and behavioural symptoms’.

MDD symptoms were assessed with the 16-item Quick Inventory Of Depressive Symptomatology (QIDS; Rush et al., 2003). The nine aggregated QIDS-items (range 0–3) were dichotomized as follows: a score of 2 or 3 (e.g. ‘I feel sad more than half the time’ and ‘I feel sad nearly all of the time’) was treated as a symptom endorsed. Following the diagnostic rule of the Diagnostic Statistical manual for Mental Disorders fifth edition (DSM-5; American Psychiatric Association, 2013) the MDD symptom cluster was considered to be present when participants endorsed at least five of nine MDD symptoms (i.e. ‘Sleep difficulties’, ‘Depressed mood’, ‘Weight gain/loss’, ‘Diminished concentration’, ‘Worthlessness’, ‘Recurrent thoughts of death/suicide’^1^, ‘Diminished interest in activities’, ‘Fatigue’, and ‘Psychomotor agitation or retardation’), with at least one of these five symptoms being either ‘Depressed mood’ or ‘Diminished interest in activities’.

PTSD symptoms as defined in DSM-5 were assessed with the 20-item PTSD Checklist for DSM-5 (Blevins, Weathers, Davis, Witte, & Domino, 2015; Boeschoten, Bakker, Jongedijk, & Olff, 2014). Each item (range 0–4) was dichotomized, treating each item rated as 2 (‘moderately’), 3 (‘quite a bit’), or 4 (‘extremely’) as a symptom endorsed. Following the DSM-5 diagnostic rule we divided the 20 items over four clusters that were dichotomized as either present or absent and, as such, used as indicators in the LCA. The ‘intrusion cluster’ was considered present when at least one B-cluster symptom was endorsed; the ‘avoidance cluster’ was considered present if at least one C-cluster symptom was endorsed; the ‘negative alterations in cognition and mood cluster’ was considered present if at least two D-cluster symptoms were endorsed, and the ‘alterations in arousal and reactivity cluster’ was considered present when at least two E-cluster symptoms were endorsed.

Cronbach’s alphas of the non-dichotomized PGD, MDD, and PTSD measure were 0.86, 0.81, and 0.93 respectively. Cronbach’s alpha of the PGD cluster ‘Cognitive, emotional, and behavioural symptoms’ was 0.85 and alphas of the PTSD B-, C-, D-, and E-Clusters were 0.83, 0.86, 0.82, and 0.83, respectively.

#### 2.3.2. Independent variables

The sociodemographic characteristics (i.e. gender, kinship to the closest deceased [0 = child or spouse, 1 = parent or sibling, 2 = other], and time since loss [in days]), disaster-related variables (i.e. number of relatives lost due to the plane crash [0 = single loss, 1 = multiple loss], number of burials organized for closest deceased relative [0 = none or multiple, 1 = one], time to confirmation of death for closest deceased relative [in days]), number of experienced life events, and a sense of unrealness were included as covariates in the analyses.

The ‘number of experienced adverse life events’ was assessed with the Life Events Scale (van der Velden, van der Burg, Steinmetz, & van den Bout, 1992). Participants were instructed to rate whether they experienced 13 events (e.g. sexual violence). A sum score was obtained of the number of experienced events.

A sense of unrealness was assessed with the 5-item Experienced Unrealness Scale (Boelen, 2010). Participants were instructed to rate to what extent they agreed with each item (e.g. ‘I have trouble believing that I will never see [–] again’) on 8-point scales (1 = ‘not at all true for me’, 8 = ‘completely true for me’). A higher score is indicative of a more pervasive sense that the loss is unreal. Psychometric properties of the scale are adequate (Boelen, 2010). Cronbach’s alpha in the current study was 0.90.

Functional impairment attributable to the plane crash was assessed with the 5-item Work and Social Adjustment Scale (de Graaf et al., 2009; Mundt, Marks, Shear, & Greist, 2002). Participants were instructed to rate on 9-point scales (0 = ‘not at all’ to 8 = ‘very severe’) to what extent they experienced impairments in functioning (e.g. social activities). A higher score is indicative of more severe impairment. Psychometric properties of this instrument are adequate (Mundt et al., 2002). Cronbach’s alpha in the current study was 0.85.

The instructions of the measures to assess PGD, PTSD, sense of unrealness, and functional impairment were adapted to refer to the disaster-related loss. We did not adapt the instruction of the MDD measure, because the original instruction of this measure does not refer to a specific event, in contrast to the PGD and PTSD measure. In addition, we aimed to measure participants’ depression levels, and we did not aim to measure the participant’s perception of depression levels related to the disaster.

### 2.4. Missing data

Because this research project was a collaborative initiative of several research institutes, not all participants filled in the same measures. Twenty-five randomly chosen participants did not complete the PTSD measure used in the current study. In addition, three other participants did also not complete the PTSD measure and/or MDD measure due to other reasons. These missing data were handled by using full maximum likelihood estimation in the LCA.

### 2.5. Statistical analyses

LCA was used to identify classes of PGD, MDD, and PTSD using Latent GOLD version 5.0 (Vermunt & Magidson, 2013). In line with clinical practice, we focused on the absence or presence of symptoms. Symptom clusters instead of individual symptoms were used as dichotomous indicators to reduce the number of indicators in the LCA. The following dichotomous indicators were modelled in order to assign participants to classes: (a) two PGD indicators (i.e. ‘separation distress’ and ‘cognitive, emotional, and behavioural symptoms’), (b) one MDD indicator, and (c) four PTSD indicators (i.e. ‘intrusion’, ‘avoidance’, ‘negative alterations in cognition and mood’, and ‘alterations in arousal and reactivity’).

First, a one-class model was estimated, followed by models with increasing numbers of classes. The optimal class-solution was selected based on the following criteria: (1) lower Sample-Size Adjusted Bayesian Information Criterion (SA-BIC) and Akaike’s Information Criterion (AIC) (i.e. frequently used to compare the fit of the models with different number of classes) (Nylund, Asparouhov, & Muthén, 2007), (2) bootstrap likelihood ratio test (BLRt) p-value < .05 (meaning a significant improvement of fit of the current solution relative to the solution with one less class) (Nylund et al., 2007), (3) higher entropy R^2^ (i.e. indication of latent class separation) (Carragher, Adamson, Bunting, & McCann, 2009), and (4) class sample size.

To test whether levels of functional impairment, PGD, MDD, and PTSD differed significantly between the classes, we separately added the sum scores of the measures to the model as covariate by using the ‘three-step approach’ implemented in Latent GOLD. In the first step of this approach a latent class model is built based on indicator variables. In the second step, participants are assigned to classes. In the third step, associations between covariates and classes are modelled, while taking into account the classification error as a result of assigning participants to classes (Vermunt, 2010).

Lastly, the sociodemographic (i.e. gender, kinship to the closest deceased, and time since loss), disaster-related variables (i.e. number of relatives lost, number of burials organized for closest deceased relative, time to confirmation of death for closest deceased relative), number of experienced life events, and a sense of unrealness were added simultaneously as covariates in the three-step approach in Latent GOLD, in order to examine which of the variables distinguished best between classes, when taking into account the shared variance between the variables.

## 3. Results

### 3.1. Participants

Table 1 shows sample characteristics. The majority of the participants were women (59.3%), highly educated (69.9%), and had lost one (32.3%) or two (34.1%) relatives. Twenty-two participants (13.2%) had lost three relatives, 31 (18.6%) four relatives, and three (1.8%) had lost five or six relatives. Ordered from closest to more distant deceased relatives, 47 participants (28.3%) had lost at least one child, 2 participants (1.2%) lost a spouse, 14 participants (8.4%) a parent, 47 (28.3%) a sibling, and 56 (33.7%) another relative or friend. The majority of the participants (80.2%) had buried remains of their closest relative once, 21 (13.0%) buried remains of the deceased on successive occasions, and 11 participants (6.8%) had not been able to bury any remains of their closest relative. Based on the reported date of birth of the deceased, the data appear to involve approximately 192 unique deceased victims. In total, 145 unique households (i.e. participants living at the same address) participated in the study.

**Table 1.** Sample characteristics.

	Total sample (*n* = 167)
***Sociodemographic variables***	
Gender, *N* (%)	
Men	68 (40.7)
Women	99 (59.3)
Age, *M* (SD)	52.49 (15.65)
Time since loss in days, *M* (SD)	343.87 (52.76)
Educational level, *N* (%)	
Primary to medium	50 (30.1)
High	116 (69.9)
Closest related deceased person was: *N* (%)	
Child or spouse	49 (29.5)
Parent or sibling	61 (36.7)
Other	56 (33.7)
***Disaster-related variables***	
Number of relatives lost, *N* (%)	
Single	54 (32.3)
Multiple	113 (67.7)
Number of burials, *N* (%)	
Once	130 (80.2)
None or more than once	32 (19.8)
Time to confirmation of death, *M* (SD)	69.91 (101.90)
***Other variables***	
Number of experienced adverse life, *M* (SD)	2.19 (1.43)
Sense of unrealness, *M* (SD)	29.04 (9.41)
***Symptom levels***	
Functional impairment, *M* (SD)	16.03 (9.50)
PGD, *M* (SD)	27.36 (7.20)
MDD, *M* (SD)	7.71 (4.78)
PTSD, *M* (SD)	19.17 (14.06)

PGD = prolonged grief disorder; MDD = major depressive disorder, PTSD = posttraumatic stress disorder. For those who experienced multiple losses, the most intimate relationship from child, through partner/spouse, to parent, to sibling, or other relative was used.

### 3.2. Latent class analysis

Based on the goodness-of-fit statistics and class sample size, for the 1–4 class solutions (see Table 2), the number of classes was chosen. The three-class solution yielded the lowest SA-BIC and AIC. Although the entropy R^2^ was lower in the three- and four-class solutions compared with the two-class solution, the significant BLRt of the three-class solution indicated that a three-class solution revealed a better fit compared with the two-class solution. The non-significant BLRt of the four-class solution showed that the four-class solution did not have a better fit compared with the three-class solution. Based on all this, we chose the more parsimonious three-class solution as optimal solution. We also examined the *p*-values of each indicator, testing the discriminative ability of the indicator. All *p*-values (except for the MDD indicator *p *= 0.28) were below 0.05, meaning that each indicator significantly contributed to the ability to discriminate between the three classes.

**Table 2.** Goodness-of-fit statistics for 1–4 class solutions.

Model	Loglikelihood	SA-BIC	AIC	BLRt (*p* =)	Entropy R^2^
1 class	−595.34	1204.34	1204.68		
2 class	−498.22	1025.72	1026.44	<.01	**0.77**
3 class	−489.19	**1023.27**	**1024.38**	**<0.05**	0.69
4 class	−483.63	1027.76	1029.25	0.28	0.69

SA-BIC = Sample-Size Adjusted Bayesian Information Criterion; AIC = Akaike’s Information Criterion; BLRt = bootstrap likelihood ratio test.

**Figure 1.**
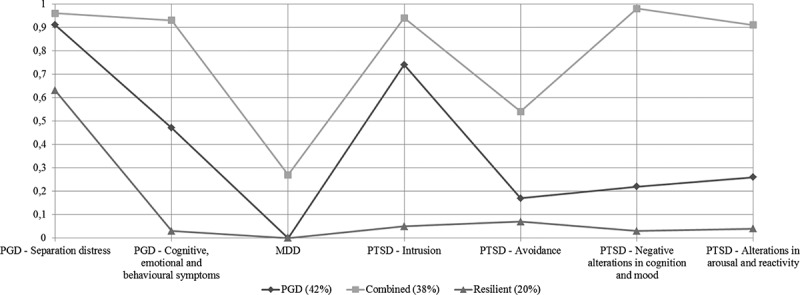
Estimated symptom probabilities for the three-class solution. PGD = prolonged grief disorder; MDD = major depressive disorder, PTSD = posttraumatic stress disorder.

Prevalence rates for the total sample and conditional probability rates with standard errors for each of the three classes are presented in Table 3. The probability rates are also displayed in Figure 1. Probability rates represent the probability of presence of the symptom cluster given the specific class (e.g. ‘Separation distress’ was present in 96% of the participants in class 3). Following the example of previous LCA research, probability rates of ≥ 0.60 represent high, ≤ 0.59 and ≥ 0.15 moderate, and < 0.15 low probability that the symptom cluster was present among the individuals in the respective class (Burstein et al., 2012; Nickerson et al., 2014).

**Table 3.** Probability of item endorsement for prolonged grief, depression, and posttraumatic stress symptoms for three-class solution.

			Overall symptom frequency		Resilient class (1) (20.0%)		PGD class (2) (41.8%)		Combined class (3) (38.2%)	
Item-number	Symptom	Cluster	N	%	Prob.	SE	Prob.	SE	Prob.	SE
*PGD symptoms*										
3	Yearning	Separation distress	145	87.3	0.63	0.11	0.91	0.05	0.96	0.03
4–12	Confusing about one’s role in life, Difficulty accepting death, Avoidance of reminders of the loss, Difficulty trusting others, Bitterness or anger, Difficulty moving on, Numbness, Feeling life is meaningless, Feeling stunned	Cognitive, emotional, and behavioural symptoms	93	55.7	0.03	0.06	0.47	0.08	0.93	0.04
*MDD symptoms*										
1–16	Sleep difficulties, Depressed mood, Weight gain/loss, Diminished concentration, Worthlessness, Recurrent thoughts of death/suicide, Diminished interest in activities, Fatigue, Psychomotor agitation or retardation	MDD	17	10.3	0.00	0.01	0.00	0.00	0.27	0.06
*PTSD symptoms*										
1–5	Intrusive memories, Disturbing dreams, Reliving, Feeling upset, Physical reactions	Intrusion symptoms	94	67.6	0.05	0.11	0.74	0.09	0.94	0.04
6–7	Avoidance of internal reminders, Avoidance of external reminders	Avoidance symptoms	40	28.8	0.07	0.09	0.17	0.07	0.54	0.07
8–14	Inability to remember aspect of the event, Negative beliefs, Self-blame, Negative mood, Diminished interest in activities, Feelings of detachment, Inability to experience positive emotions	Negative alterations in cognition and mood	65	46.8	0.03	0.06	0.22	0.08	0.98	0.03
15–20	Irritable, Taking risks, Being superalert, Feeling jumpy, Concentration problems, Sleep disturbance	Alterations in arousal and reactivity	64	46.0	0.04	0.06	0.26	0.07	0.91	0.05

PGD = prolonged grief disorder; MDD = major depressive disorder; PTSD = posttraumatic stress disorder; Prob. = probability estimate; SE = standard error

Class 1 (denoted as the Resilient class; 20.0%) was characterized by a low probability of presence of PGD, MDD, and PTSD symptom clusters. Class 2 (denoted as the PGD class; 41.8%) was characterized by a moderate to high probability of presence of the two PGD symptoms clusters and the PTSD ‘intrusion cluster’. Class 3 (coined the Combined class; 38.2%) was characterized by a high probability that both PGD symptom clusters and three PTSD symptom clusters were present. In addition, both in the Resilient class and in the PGD class, MDD (i.e. endorsement of at least five of nine MDD symptoms) was not present among the participants, while in the Combined class, MDD was present in 26.6% of participants.

### 3.3. Differences in functional impairment, PGD, MDD, and PTSD between the classes

Table 4 shows the results of tests for differences between the classes as function of the sum scores of functional impairment, PGD, MDD, or PTSD levels. The Resilient class was used as reference class. In short, levels of functional impairment, PGD, and PTSD were significantly (*p *< 0.05) higher in the PGD class and in the Combined class compared with the Resilient class. The PGD class did not significantly differ from the Resilient class with respect to MDD levels, but the Combined class reported significantly (*p *< 0.05) higher MDD levels compared with the Resilient class. Based on the 95% confidence intervals (CIs), the Combined class also reported significantly higher levels of functional impairment, PGD, MDD, and PTSD compared with the PGD class.

**Table 4.** Parameter estimates for the latent class model with levels of functional impairment, PGD, MDD, or PTSD as covariate.

	PGD vs. Resilient class			Combined vs. Resilient class			
Covariates	B	SE (B)	95% CI	B	SE (B)	95% CI	*p*
Functional impairment	0.17	0.06	0.05–0.29	0.35	0.07	0.21–0.49	<.001
PGD	0.50	0.16	0.18–0.81	0.93	0.16	−0.62–1.24	<.001
MDD	0.14	0.10	−0.06–0.34	0.65	0.14	0.38–0.92	<.001
PTSD	0.40	0.14	0.13–0.67	1.02	0.18	0.67–1.37	<.001

95% CI = 95% confidence interval; PGD = prolonged grief disorder; MDD = major depressive disorder; PTSD = posttraumatic stress disorder; SE = standard error

### 3.4. Correlates of class membership

Table 5 summarizes tests for differences between the classes in terms of sociodemograpic variables, disaster-related variables, number of experienced adverse life events, and sense of unrealness when entered simultaneously into the model. The Resilient class was used as reference class. Sense of unrealness was significantly associated with class membership. Participants in the PGD class (B = 0.13 (95% CI = 0.03–0.23), *p *< 0.05) and in the Combined class (B = 0.25 (95% CI = 0.13–0.37), *p *< 0.05) scored significantly higher on sense of unrealness than individuals in the Resilient class. Unrealness did not differ between individuals in the Combined class and PGD class. None of the other variables were significantly associated with class membership.

**Table 5.** Parameter estimates for the latent class model with covariates.

	PGD vs. Resilient class			Combined vs. Resilient class			
Covariates	B	SE (B)	95% CI	B	SE (B)	95% CI	*p*
Gender	0.93	0.78	−0.60–2.46	0.56	0.83	−2.19–1.07	0.48
Times since loss in days	0.00	0.00	0.00–0.00	−0.01	0.01	−0.03–0.01	0.23
Closest deceased person was^a^							0.29
Child or spouse vs. other	0.41	1.15	−1.84–2.66	−0.02	1.28	−2.53–2.49	
Parent or sibling vs. other	−0.29	0.84	−1.94–1.36	0.77	0.88	−0.95–2.49	
Number of relatives lost^a^	−0.79	0.77	−2.30–0.72	−0.30	0.77	−1.81–1.21	0.57
Number of burials^a^	−0.06	0.94	−1.90–1.78	1.19	0.88	−0.53–2.91	0.16
Time to confirmation of death	−0.00	0.01	−0.02–0.02	0.00	0.01	−0.02–0.02	0.22
Number of experienced adverse life	−0.22	0.27	−0.75–0.31	0.22	0.24	−0.25–0.69	0.37
Sense of unrealness	0.13	0.05	0.03–0.23	0.25	0.06	0.13–0.37	< 0.001

The categorical variables were coded as follows: gender (0 = women, 1 = men), closest deceased person (0 = child/spouse, 1 = parent/sibling, 2 = other), number of relatives lost (0 = single loss, 1 = multiple loss), and number of burials (0 = none or multiple, 1 = one); 95% CI = 95% confidence interval; PGD = prolonged grief disorder. ^a^Kinship to the deceased, number of relatives lost, and number of burials of the closest deceased were more broadly categorized to prevent empty cells. For those who experienced multiple losses, the most intimate relationship from child, through partner/spouse, to parent, to sibling, or other relative was used.

## 4. Discussion

In the current study we sought to identify distinct subgroups among disaster-bereaved individuals, based on presence of symptom clusters of PGD, MDD, and PTSD, using LCA. Three subgroups were identified: (a) a Resilient class characterized by a low probability of presence of PGD, MDD, and PTSD symptom clusters, (b) a PGD class characterized by a moderate to high probability of presence of both symptom clusters of PGD, and (c) a Combined class characterized by a high probability of presence of both symptom clusters of PGD, three of the four PTSD symptom clusters, and a moderate probability of presence of MDD. In line with two previous LCA studies in people confronted with traumatic loss (Boelen et al., 2016; Nickerson et al., 2014), our results indicate that subgroups of individuals can be distinguished based on the presence vs. absence of psychopathology symptoms. All three studies showed a Resilient class, PGD class, and a class with combined symptoms. The consistency in the findings across the studies supports that PGD is distinguishable from MDD and PTSD, which has also been found previously with factor analyses (Prigerson, Bierhals, Kasl, & Reynolds, 1996). Unlike Nickerson et al. (2014) our findings did not show a distinct PTSD group. One plausible explanation for this difference may be that Nickerson et al. (2014) studied refugees who were confronted with a loss but also with other potentially traumatic refugee-related events. In line with LCA studies among bereaved and traumatized individuals (Boelen et al., 2016; Galatzer-Levy, Nickerson, Litz, & Marmar, 2013), we could not distinguish a class characterized by a high probability of MDD symptoms. This suggests that MDD is predominantly present in combination with other symptoms among people confronted with a traumatic loss or other traumatic event.

We believe it is important to emphasize that although 80.0% of the participants were assigned to classes characterized by psychopathology symptom clusters, this does not imply that all these participants meet the diagnostic criteria for a psychiatric disorder. Looking at the results, we see, for instance, that PGD symptom clusters were present in only 53% of the participants, which is consistent with other studies among disaster-bereaved individuals (Bonanno et al., 2006; Kristensen et al., 2010). A restrained approach towards offering psychological support to all disaster-bereaved individuals therefore seems warranted.

With respect to the correlates of class membership, we found that the extent of experiencing a sense of unrealness distinguished best between the Resilient class and the two psychopathology classes, when taking into account the effect of other variables. However, the extent of experiencing a sense of unrealness did not differ between individuals in the Combined and PGD class. In other words, higher levels of a sense of unrealness differentiate individuals with resilient responses from individuals with elevated PGD symptoms as well as individuals with elevated PGD, MDD, and PTSD levels, which is in line with previous research (Boelen, 2010). However, unrealness does not differentiate individuals with ‘solely’ PGD from individuals with elevated PGD, MDD, and PTSD levels. This may indicate that a sense of unrealness is mainly associated with PGD symptoms and to a lesser extent with MDD and PTSD symptoms. Furthermore, other variables may determine whether post-disaster bereaved individuals develop PGD with comorbid symptoms, such as previous psychiatric disorders prior to the loss (Simon et al., 2007), media exposure (Neria et al., 2007), and negative cognitions (Boelen et al., 2016).

With respect to the disaster-related variables we registered, our findings indicated that deviating funeral rituals (i.e. not being able to bury a loved one or having multiple burials for the same deceased instead of burying remains of the loved one once) did not distinguish between the different classes. Although previous research advocated the protective role of performing traditional grief rituals (Castle & Phillips, 2003; Stammel et al., 2013) our findings were not in line with this notion. One possible explanation for this may be that the majority of our sample experienced multiple losses and we only included the number of burials for the closest deceased relative in our analyses. The number of burials for the other deceased relatives may therefore confound the results.

In contrast to the findings in tsunami-bereaved individuals (Kristensen et al., 2010) time to confirmation of death was not related to psychopathology in the current study. One explanation for this difference may be associated with the nature of the disaster. The tsunami-bereaved individuals may still have experienced hope that the missing loved one survived the tsunami as long as death was not confirmed. Previous research has been shown that maintaining hope that a disappeared loved one is still alive is associated with increased PGD levels (Heeke, Stammel, & Knaevelsrud, 2015). In the current sample, it was impossible that any of the passengers of flight MH17 survived the crash.

Lastly, experiencing single versus multiple losses was also not a distinguishable feature of resilient and more disturbed subgroups of bereaved individuals. Previous studies also showed that number of losses is unrelated to psychopathology levels (Li et al., 2015; Stammel et al., 2013).

The fact that we found no clinical correlates of class membership (apart from unrealness) limits the pragmatic value of our findings. That is, our analyses do not shed light on sociodemographic and disaster-related correlates of classes that are useful to predict which people will be in resilient or problematic classes. Future research is needed to further evaluate sociodemographic, disaster-related, and other (e.g. coping) variables possibly associated with different bereaved subgroups confronted with unnatural loss.

Our LCA results may generate useful information about symptomatology in people confronted with a potential traumatic loss of a loved one. The probability that the PGD symptom cluster ‘Separation distress’ was present was high in all subgroups. Therefore, the intensity of separation distress is relatively uninformative in distinguishing adaptive from maladaptive responses to unnatural loss. The probability that the PTSD ‘Intrusion’ symptom cluster was present was high in both psychopathology subgroups, indicating overlap between PGD and PTSD. In contrast, the MDD symptom cluster was relatively uncommon in the current sample and may therefore be less relevant as a target of treatment of disaster-bereaved individuals. These findings are in line with previous LCA results (Boelen et al., 2016; Nickerson et al., 2014). With respect to bereaved individuals in need of professional support, those with ‘solely’ PGD may benefit most from cognitive behavioural therapy focused on the grieving process (Currier, Holland, & Neimeyer, 2010; Rosner, Pfoh, & Kotoučová, 2011). Individuals with PGD, MDD, and PTSD symptoms may benefit most from eclectic therapy that targets a comorbid symptom profile (Smid et al., 2015).

Several limitations of the current study need to be taken into account while interpreting the results. Firstly, we chose to select PGD symptoms (as proposed by Prigerson et al., 2009), in order to compare our results with previous LCA studies in bereaved samples (Boelen et al., 2016; Nickerson et al., 2014). Our results may therefore not be generalizable to studies using the persistent complex bereavement disorder (PCBD) DSM-5 criteria. However, a recent study showed high communalities between PGD and PCBD; apart from the difference in timeframe (i.e. PGD six months and PCBD 12 months post-loss) they only differ in semantic terms (Maciejewski, Maercker, Boelen, & Prigerson, 2016). One may argue that our findings may not be indicative of presence of distorted grief symptoms as mean time since loss of the current sample is less than one year. However, the 12-months criterion of PCBD is not empirically based. Multiple studies have shown that abnormal grief can be distinguished from normal grief six-months post-loss (Maciejewski et al., 2016; Prigerson et al., 2009; Shear et al., 2011). Secondly, although no strict sample size guidelines for conducting LCA are available (Wurpts & Geiser, 2014), we are aware that our sample size is relatively small and we therefore could only use a limited number of indicators. We also used dichotomized indicators based on symptom clusters that are used for diagnostic purposes in clinical practice, which may lead to less precision in detecting meaningful classes (van Loo, de Jonge, Romeijn, Kessler, & Schoevers, 2012). While our results offer insight in the different combinations of symptom clusters, which can occur in post-disaster bereaved individuals, it is important to keep in mind that the dimensionality of MDD and PTSD are subject to discussion (cf. Armour, Fried, Deserno, Tsai, & Pietrzak, 2016; Fried et al., 2016). Furthermore, other statistical models, such as mixture models or network analyses, could be used in future research to generate further insight in the representation and coherence of symptoms among disaster-bereaved individuals individuals (cf. Elhai, Naifeh, Forbes, Ractliffe, & Tamburrino, 2011; McNally et al., 2015). Thirdly, we did not account for the nested structure of the data in the analyses (i.e. 13.2% of the participants shared their household with at least one other participant). The observations per individual may be not completely independent, and as a result, be biased. Given that we observed relatively low number of level-1 units (i.e. participants) per level-2 unit (i.e. households), it seems unlikely that a multilevel approach would yield meaningful differences in the results. Fourthly, the results may not be generalizable to all disaster-bereaved individuals, due to the use of a self-selected sample. Lastly, self-rated questionnaires were used, which may lead to an overestimation of symptom levels (Engelhard et al., 2007).

In conclusion, LCA revealed three subgroups of post-disaster bereaved individuals based on presence of PGD, MDD, and PTSD symptom clusters; a Resilient, PGD, and Combined class. This is consistent with previous LCA research in bereaved individuals (Boelen et al., 2016; Nickerson et al., 2014). A sense of unrealness was the strongest distinguishing feature of the subgroups.

## Note

1. We omitted the last answer category of this item for ethical reasons.

## Highlights

- We examined prolonged grief disorder (PGD), major depressive disorder (MDD), and posttraumatic stress disorder (PTSD) among people confronted with losses due to the Ukrainian plane crash
- Latent class analysis revealed a Resilient class (20.0%), a PGD class (41.8%), and a combined PGD, MDD, and PTSD class (38.2%)
- Endorsement of ‘a sense of unrealness’ differentiated the Resilient class from the two psychopathology classes

## References

[CIT0001] American Psychiatric Association (2013). *Diagnostic and statistical manual of mental disorders*.

[CIT0002] Armour C., Contractor A., Elhai J. D., Stringer M., Lyle G., Forbes D., Richardson J. D. (2015). Identifying latent profiles of posttraumatic stress and major depression symptoms in Canadian veterans: Exploring differences across profiles in health related functioning. *Psychiatry Research*.

[CIT0003] Armour C., Fried E. I., Deserno M. K., Tsai J., Pietrzak R. H. (2016). A Network Analysis of DSM-5 posttraumatic stress disorder symptoms and correlates in U.S. military veterans. *Journal Of Anxiety Disorders*.

[CIT0004] Blevins C. A., Weathers F. W., Davis M. T., Witte T. K., Domino J. L. (2015). The posttraumatic stress disorder checklist for DSM-5 (PCL-5): Development and initial psychometric evaluation. *Journal of Traumatic Stress*.

[CIT0005] Boelen P. A. (2010). A sense of ‘unrealness’ about the death of a loved-one: An exploratory study of its role in emotional complications among bereaved individuals. *Applied Cognitive Psychology*.

[CIT0006] Boelen P. A., Reijntjes A., Djelantik M., Smid G. E. (2016). Prolonged grief and depression after unnatural loss: Latent class analyses and cognitive correlates. *Psychiatry Research*.

[CIT0007] Boelen P. A., Smid G. E. (in press). The Traumatic Grief Inventory Self Report Version (TGI-SR): Introduction and preliminary psychometric evaluation. *Journal of Loss and Trauma*.

[CIT0008] Boelen P. A., van de Schoot R., van den Hout M. A., de Keijser J., van den Bout J. (2010). Prolonged Grief Disorder, depression, and posttraumatic stress disorder are distinguishable syndromes. *Journal of Affective Disorders*.

[CIT0009] Boelen P. A., van den Hout M. A., van den Bout J. (2006). A cognitive-behavioral conceptualization of complicated grief. *Clinical Psychology: Science and Practice*.

[CIT0010] Boeschoten M. A., Bakker A., Jongedijk R. A., Olff M. (2014). *PTSS checklist voor de DSM-5 (PCL-5)*.

[CIT0011] Bonanno G. A., Galea S., Bucciarelli A., Vlahov D. (2006). Psychological resilience after disaster: New york city in the aftermath of the september 11th terrorist attack. *Psychological Science*.

[CIT0012] Burstein M., Georgiades K., Lamers F., Swanson S. A., Cui L., He J.-P., Merikangas K. R. (2012). Empirically derived subtypes of lifetime anxiety disorders: Developmental and clinical correlates in U.S. adolescents. *Journal of Consulting and Clinical Psychology*.

[CIT0013] Carragher N., Adamson G., Bunting B., McCann S. (2009). Subtypes of depression in a nationally representative sample. *Journal of Affective Disorders*.

[CIT0014] Castle J., Phillips W. L. (2003). Grief rituals: Aspects that facilitate adjustment to bereavement. *Journal of Loss & Trauma*.

[CIT0015] Cloitre M., Garvert D., Weiss B., Carlson E., Bryant R. (2014). Distinguishing PTSD, Complex PTSD, and Borderline Personality Disorder: A latent class analysis. *European Journal Of Psychotraumatology*.

[CIT0016] Contractor A. A., Elhai J. D., Fine T. H., Tamburrino M. B., Cohen G., Shirley E., Calabrese J. R. (2015). Latent profile analyses of posttraumatic stress disorder, depression and generalized anxiety disorder symptoms in trauma-exposed soldiers. *Journal Of Psychiatric Research*.

[CIT0017] Currier J. M., Holland J. M., Neimeyer R. A. (2010). Do CBT-based interventions alleviate distress following bereavement? A review of the current evidence. *International Journal of Cognitive Therapy*.

[CIT0018] de Graaf L. E., Gerhards S. A., Arntz A., Riper H., Metsemakers J. F., Evers S. M., Huibers M. J. (2009). Clinical effectiveness of online computerised cognitive-behavioural therapy without support for depression in primary care: Randomised trial. *The British Journal of Psychiatry: the Journal of Mental Science*.

[CIT0019] Dutch Safety Board (2015). *MH17 crash*.

[CIT0020] Elhai J. D., Naifeh J. A., Forbes D., Ractliffe K. C., Tamburrino M. (2011). Heterogeneity in clinical presentations of posttraumatic stress disorder among medical patients: Testing factor structure variation using factor mixture modeling. *Journal of Traumatic Stress*.

[CIT0021] Engelhard I. M., van den Hout M. A., Weerts J., Arntz A., Hox J. J., McNally R. J. (2007). Deployment-related stress and trauma in dutch soldiers returning from iraq. prospective study. *The British Journal of Psychiatry: the Journal of Mental Science*.

[CIT0022] Fried E. I., van Borkulo C. D., Epskamp S., Schoevers R. A., Tuerlinckx F., Borsboom D. (2016). Measuring depression over time. Or not? Lack of unidimensionality and longitudinal measurement invariance in four common rating scales of depression. *Psychological Assessment*.

[CIT0023] Galatzer-Levy I. R., Nickerson A., Litz B. T., Marmar C. R. (2013). Patterns of lifetime PTSD comorbidity: A latent class analysis. *Depression and Anxiety*.

[CIT0024] Galea S., Nandi A., Vlahov D. (2005). The epidemiology of post-traumatic stress disorder after disasters. *Epidemiologic Reviews*.

[CIT0025] Heeke C., Stammel N., Knaevelsrud C. (2015). When hope and grief intersect: Rates and risks of prolonged grief disorder among bereaved individuals and relatives of disappeared persons in colombia. *Journal of Affective Disorders*.

[CIT0026] Johannesson K. B., Lundin T., Hultman C. M., Fröjd T., Michel P. (2011). Prolonged grief among traumatically bereaved relatives exposed and not exposed to a tsunami. *Journal of Traumatic Stress*.

[CIT0027] Kristensen P., Weisaeth L., Heir T. (2010). Predictors of complicated grief after a natural disaster: A population study two years after the 2004 south-east asian tsunami. *Death Studies*.

[CIT0028] Kristensen P., Weisaeth L., Hussain A., Heir T. (2015). Prevalence of psychiatric disorders and functional impairment after loss of a family member: A longitudinal study after the 2004 Tsunami. *Depression and Anxiety*.

[CIT0029] Li J., Chow A. Y. M., Shi Z., Chan C. L. W. (2015). Prevalence and risk factors of complicated grief among sichuan earthquake survivors. *Journal of Affective Disorders*.

[CIT0030] Maciejewski P. K., Maercker A., Boelen P. A., Prigerson H. G. (2016). “Prolonged grief disorder” and “persistent complex bereavement disorder”, but not “complicated grief”, are one and the same diagnostic entity: An analysis of data from the Yale bereavement study. *World Psychiatry*.

[CIT0031] Maercker A., Znoj H. (2010). The younger sibling of PTSD: Similarities and differences between complicated grief and posttraumatic stress disorder. *European Journal of Psychotraumatology*.

[CIT0032] McNally R. J., Robinaugh D. J., Wu G. W. Y., Wang L., Deserno M. K., Borsboom D. (2015). Mental disorders as causal systems: A network approach to posttraumatic stress disorder. *Clinical Psychological Science*.

[CIT0033] Mundt J. C., Marks I. M., Shear M. K., Greist J. H. (2002). The work and social adjustment scale: A simple measure of impairment in functioning. *The British Journal of Psychiatry: the Journal of Mental Science*.

[CIT0034] Neria Y., Gross R., Litz B., Maguen S., Insel B., Seirmarco G., Marshall R. D. (2007). Prevalence and psychological correlates of complicated grief among bereaved adults 2.5–3.5 years after september 11th attacks. *Journal of Traumatic Stress*.

[CIT0035] Nickerson A., Liddell B. J., Maccallum F., Steel Z., Silove D., Bryant R. A. (2014). Posttraumatic stress disorder and prolonged grief in refugees exposed to trauma and loss. *BMC Psychiatry*.

[CIT0036] Nylund K. L., Asparouhov T., Muthén B. O. (2007). Deciding on the number of classes in latent class analysis and growth mixture modeling: A monte carlo simulation study. *Structural Equation Modeling: A Multidisciplinary Journal*.

[CIT0037] Perlman S. E., Friedman S., Galea S., Nair H. P., Erős-Sarnyai M., Stellman S. D., Greene C. M. (2011). Short-term and medium-term health effects of 9/11. *The Lancet*.

[CIT0038] Prigerson H. G., Bierhals A. J., Kasl S. V., Reynolds C. F., Shear M. K., Newsom J. T., Jacobs S. (1996). Complicated grief as a disorder distinct from bereavement-related depression and anxiety: A replication study. *The American Journal of Psychiatry*.

[CIT0039] Prigerson H. G., Horowitz M. J., Jacobs S. C., Parkes C. M., Aslan M., Goodkin K., Maciejewski P. K. (2009). Prolonged grief disorder: Psychometric validation of criteria proposed for DSM-V and ICD-11. *Plos Medicine*.

[CIT0040] Rosner R. (2015). Prolonged grief: Setting the research agenda. *European Journal of Psychotraumatology*.

[CIT0041] Rosner R., Pfoh G., Kotoučová M. (2011). Treatment of complicated grief. *European Journal of Psychotraumatology*.

[CIT0042] Rush A. J., Trivedi M. H., Ibrahim H. M., Carmody T. J., Arnow B., Klein D. N., Keller M. B. (2003). The 16-item quick inventory of depressive symptomatology (QIDS), clinician rating (QIDS-C), and self-report (QIDS-SR): A psychometric evaluation in patients with chronic major depression. *Biological Psychiatry*.

[CIT0043] Shear M. K., Simon N., Wall M., Zisook S., Neimeyer R., Duan N., Keshaviah A. (2011). Complicated grief and related bereavement issues for DSM-5. *Depression and Anxiety*.

[CIT0044] Simon N. M., Shear K. M., Thompson E. H., Zalta A. K., Perlman C., Reynolds C. F., Silowash R. (2007). The prevalence and correlates of psychiatric comorbidity in individuals with complicated grief. *Comprehensive Psychiatry*.

[CIT0045] Smid G. E., Kleber R. J., De La Rie S. M., Bos J. B., Gersons B. P., Boelen P. A. (2015). Brief eclectic psychotherapy for traumatic grief (BEP-TG): Toward integrated treatment of symptoms related to traumatic loss. *European Journal of Psychotraumatology*.

[CIT0046] Stammel N., Heeke C., Bockers E., Chhim S., Taing S., Wagner B., Knaevelsrud C. (2013). Prolonged grief disorder three decades post loss in survivors of the khmer rouge regime in cambodia. *Journal of Affective Disorders*.

[CIT0047] van der Velden P. G., van der Burg S., Steinmetz C. H. D., van den Bout J. (1992). *Slachtoffers van bankovervallen* [Victims of back robberies].

[CIT0048] van Loo H. M., de Jonge P., Romeijn J.-W., Kessler R. C., Schoevers R. A. (2012). Data-driven subtypes of major depressive disorder: A systematic review. *BMC Medicine*.

[CIT0049] Vermunt J. K. (2010). Latent class modeling with covariates: Two improved three-step aproaches. *Political Analysis*.

[CIT0050] Vermunt J. K., Magidson J. (2013). *Latent GOLD 5.0 upgrade manual*.

[CIT0051] Wurpts I. C., Geiser C. (2014). Is adding more indicators to a latent class analysis beneficial or detrimental? Results of a monte-carlo study. *Frontiers in Psychology*.

